# Type III Duane Retraction Syndrome and Monocular Elevation Deficiency, Simultaneous Presentation in a 16-year-old girl

**DOI:** 10.22336/rjo.2024.79

**Published:** 2024

**Authors:** Hajar Farvardin, Majid Farvardin, Kamran Zare

**Affiliations:** Poostchi Ophthalmology Research Center, Department of Ophthalmology, School of Medicine, Shiraz University of Medical Sciences, Shiraz, Iran

**Keywords:** Type III Duane Retraction Syndrome, Monocular elevation deficiency, congenital ophthalmic disorders, DRS = Duane Retraction Syndrome, MED = Monocular Elevation Deficiency, BCVA = Best Corrected Visual Acuity, PD = Prism Diopter, XT = Exotropia, HOT = Hypotropia, FDT = Forced Duction Test

## Abstract

Duane Retraction Syndrome (DRS) and Monocular Elevation Deficiency (MED) are two distinct congenital strabismus entities that have not been reported simultaneously until now. Thirty percent of individuals with DRS present with various congenital ocular and non-ocular anomalies.

A 16-year-old girl presented with right eye ptosis and ocular deviation since birth. The patient’s hypotropia increased in the upward gaze. She also exhibited small-angle exotropia in the primary position, along with the limitation of both abduction and adduction. The patient had globe retraction and increased ptosis in adduction. Strabismus manifestation in the patient was consistent with concurrent type III DRS and MED in the right eye.

## Introduction

Duane Retraction Syndrome (DRS) is a congenital ocular movement disorder that causes limitation of abduction and/or adduction, along with narrowing of the palpebral fissure, globe retraction, and leash phenomenon on adduction [1]. The prevalence of DRS is 1/1000 in the general population and 1% to 5% among all strabismus cases [2]. Duane Retraction Syndrome is classified into three types based on Huber’s classification. DRS type I is characterized by poor eye abduction and primary position esotropia, while DRS type II shows poor eye adduction and exotropia. Both limitation of abduction and adduction defines DRS type III and may present with primary position esotropia, exotropia, or orthotropia [3]. Apart from this classification, dividing DRS patients based on their primary position horizontal deviation into eso-DRS, exo-DRS, and ortho-DRS is more straightforward, especially for surgical planning [3,4].

Monocular Elevation Deficiency (MED) is characterized by unilateral limitation in upward gaze. The prevalence of MED is about 0.5% among strabismic patients. Clinical features include hypotropia of the involved eye and elevation deficit, which may be more pronounced in abduction, along with ipsilateral ptosis and pseudoptosis [5]. MED is classified into three types based on its underlying mechanism: restriction of the inferior rectus muscle, innervation deficiency of the superior rectus muscle, and combined form. Considering MED’s heterogeneous etiology and pathogenesis, treating the condition with a single surgical method is difficult. Therefore, different surgical modalities must achieve the desired outcome [5].

Thirty percent of patients with DRS are associated with other congenital ocular and non-ocular anomalies [2]. To our knowledge, this case report is the first to describe the concurrent presentation of DRS and MED in the ophthalmologic literature.

## Case presentation

### Patient’s information

This case study followed the principles outlined in the Declaration of Helsinki, and informed consent was obtained from the patient and her parents. A 16-year-old girl was referred to our strabismus clinic with right eye ptosis and limitation of ocular motion since birth. The patient was born to a natural birth delivery at the gestational age of 38 weeks with a birth weight of 3150 grams. Her developmental stages followed a regular course, and she denied any underlying diseases. Additionally, the patient mentioned no history of strabismus or ptosis in her family members. Previous neurologic assessments, including a brain computed tomography scan, were all normal.

### Patient’s physical examination

The best corrected visual acuity (BCVA) was 20/30 in the right eye and 20/20 in the left eye, which was her dominant eye. The cycloplegic refraction was -0.5-0.25*176 in the right eye and -0.75-0.5*173 in the left. The stereoacuity checked using the Titmus stereoacuity test was approximately 80 seconds of arc. The relative afferent pupillary defect was bilateral negative, and pupils reacted symmetrically to the light on both sides. Slit lamp biomicroscopy and dilated fundus examination were normal as well. The intraocular pressure was 15 mm Hg and 16 mm Hg in the right and left eye, respectively.

**Fig. 1:**
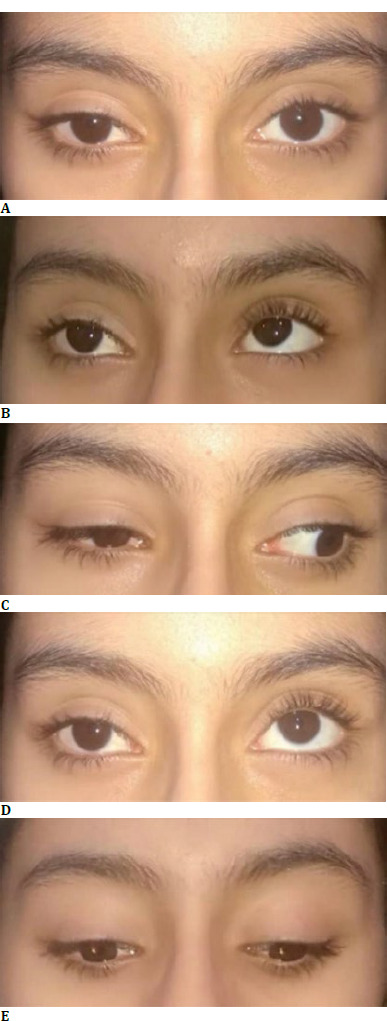
Cardinal gazes of the patient. **A**. Primary Position: Right eye upper eyelid ptosis, Right eye hypotropia, and exotropia; **B**. Right (and slightly upward) Gaze: Decreased ptosis and improved right-side palpebral fissure height, limitation of abduction in the right eye, mild limitation of adduction in the left eye; **C**. Left gaze: Aggravated ptosis and right-side globe retraction on adduction, limitation of adduction in the right eye; **D**. Up-gaze: Severe limitation of elevation in the right eye; **E**. Down-gaze: Less pronounced ptosis, no restriction of infraduction

Ocular deviation was measured using a simultaneous prism cover test. In the primary position, the patient had 12 Prism Diopter (PD) exotropia (XT) and 5 PD hypotropia (HOT) in the right eye. Right eye ptosis was also evident with MRD1 of 3.5 mm compared to 5 mm on the other side. Extraocular movement evaluation showed severe limitation of abduction and moderate limitation of adduction in the right eye. The forced duction test (FDT) in gaze to the right was positive for the right eye. The right eye’s palpebral fissure height increased in the right gaze, and the patient’s ptosis became less noticeable. Conversely, the right eye’s palpebral fissure height decreased in adduction, and the patient’s ptosis was aggravated (induced ptosis). The right eye also detected globe retraction in adduction, accompanied by a mild upshoot. Right eye hypotropia was increased from 5 PD in the primary position to 25 PD in the upward gaze. A slightly positive FDT accompanied the right eye’s limitation of elevation. Bell’s phenomenon was also significantly lower in the right eye. Palpebral fissure height was symmetric on both sides in the inferior gaze. The horizontal deviation was 12 PD of exotropia in both superior and inferior gazes, and no pattern was detected. At the time of examination, the Bielschowsky head tilt test did not make any changes in the examination, and no compensatory face turn was observed (**Fig. 1 A-E**).

### Diagnostic Assessment

Based on the mentioned findings, the patient’s diagnosis of type III Duane retraction syndrome plus Monocular elevation deficiency was determined simultaneously.

## Discussion

Duane retraction syndrome is the most common disease in the spectrum of congenital cranial dysinnervation disorders. Different mechanical, innervational, and central nervous system anomalies were previously mentioned as possible underlying mechanisms in patients with DRS [1-3]. In nearly 10% of the affected individuals, the condition might be familial, which includes an autosomal dominant mode of inheritance in associated syndromes. It was hypothesized that females would be more susceptible to expressing the responsible genes for manifesting DRS [2]. Approximately 30% of DRS patients present simultaneous congenital ocular and non-ocular anomalies. Previous systemic abnormalities reported in the literature included preauricular tags, deafness, cleft palate, facial asymmetry, cardiac anomalies, renal anomalies, limb and vertebral anomalies, imperforate anus, and microcephaly. Simultaneous ocular anomalies in the anterior segment have been reported, such as cataracts, iris coloboma, keratoconus, microcornea, and epibulbar dermoid. Various posterior segment anomalies of the optic disc and some neuro-ophthalmologic manifestations, such as the Marcus-Gunn Jaw Winking phenomenon and Horner’s syndrome, have also been reported [2]. Despite numerous ophthalmic associations of DRS patients, to the best of our knowledge, the concurrent presentation of DRS and MED has not yet been reported.

Horizontal duction deficits in the involved eye mainly characterize DRS. Anomalous vertical movements such as upshoot and downshoot on adduction frequently occur in DRS patients due to co-contraction of both medial and lateral rectus muscles [1-3]. However, vertical ductions are rarely limited in this condition. Vertical retraction syndrome was once reported in Chinese literature as a rare clinical entity [2]. Three cases of type III DRS with primary position hypertropia (probably due to superior rectus muscle hypoplasia) and one patient with simultaneous bilateral DRS and dissociative vertical deviation were previously reported in the literature [6,7]. The current case report presents the first concurrent presentation of type III DRS and MED in a 16-year-old girl.

The patient in our report presented with right eye XT and HOT in the primary position, combined with severe limitation of abduction, adduction, and elevation in the affected eye. There are a few possible differential diagnoses for the mentioned ocular deviation. Congenital fibrosis of the extraocular muscles is a severe form of congenital strabismus with restricted horizontal and vertical movements and severe ptosis. However, this patient’s unilateral presentation and severe globe retraction on adduction are against this diagnosis. Third nerve palsy, which may simulate the manifestations of MED, is another possible differential diagnosis in the mentioned case. However, the patient’s limitation of abduction and globe retraction on adduction are not justifiable with the diagnosis of oculomotor nerve palsy.

## Conclusion

It has been found that nearly 30% of patients with Duane Retraction Syndrome may also have other congenital ocular and systemic anomalies. In this case report, a 16-year-old girl was diagnosed with both DRS and MED, highlighting the importance of a comprehensive physical examination that includes a complete ophthalmic evaluation.
